# Cardiovascular Disease Prevention During the COVID-19 Pandemic: Lessons Learned and Future Opportunities

**DOI:** 10.14797/mdcvj.210

**Published:** 2021-09-24

**Authors:** Eamon Duffy, Michael Chilazi, Miguel Cainzos-Achirica, Erin D. Michos

**Affiliations:** 1 Johns Hopkins University School of Medicine, Baltimore, Maryland, US; 2 Houston Methodist DeBakey Heart & Vascular Center, Houston Methodist Hospital, Houston, Texas, US; 3 Center for Outcomes Research, Houston Methodist, Houston, Texas, US; 4 Welch Center for Prevention, Epidemiology and Clinical Research, Johns Hopkins University, Baltimore, Maryland, US

**Keywords:** cardiovascular disease, COVID-19, pandemic, prevention

## Abstract

The coronavirus disease 2019 (COVID-19) pandemic has been the defining healthcare issue since its outbreak, consuming healthcare systems and disrupting all aspects of human life throughout 2020 and continuing through 2021. When reviewing cardiovascular disease (CVD) prevention throughout the COVID-19 pandemic, the first tendency may be to focus on the negative disruption. Months of quarantine, isolation, and missed healthcare visits or delayed care may have exacerbated the epidemic of CVD in the United States. Looking back, however, perhaps it wasn’t a lost year as much as a health crisis that better prepared us for the battle to improve cardiovascular health. The pandemic brought new platforms for interacting with patients eager to engage, presenting a unique opportunity to reset how we approach preventive care.

In this review, we discuss what the pandemic has taught us about caring for those vulnerable patients who were most afflicted—older adults, persons of color, and people facing adverse socioeconomic circumstances—and who continue to be impacted by CVD. We also identify opportunities for enhanced CVD prevention now boosted by the overnight adoption of telemedicine and other innovative cardiac care models. Lastly, we discuss how the COVID-19 pandemic has motivated physicians and patients alike to prioritize our health above all else, if only transiently, and how we can leverage this increased health awareness and investment into long-term, meaningful disease prevention.

## Introduction

Since its outbreak in 2020, the coronavirus disease 2019 (COVID-19) pandemic has been the defining healthcare issue consuming healthcare systems and disrupting all aspects of human life. Almost overnight, healthcare systems shifted resources and priorities toward the care of patients with COVID-19 and the prevention of viral spread throughout communities. By October 2020, COVID-19 had become the third-leading cause of death in the United States for adults aged 45 to 84 and the second-leading cause of death for those aged 85 years and older.^1^

With the connection between the severe acute respiratory syndrome coronavirus 2 (SARS-CoV-2) and cardiovascular disease (CVD) already well established, CVD featured prominently throughout the pandemic as well. Indeed, CVD^2^ and its risk factors—obesity, diabetes mellitus, and hypertension^3,4,5^—were identified early on as common comorbidities among patients hospitalized with COVID-19 and as markers for more severe illness and death.^6^ As a result, vast initial research efforts were focused on understanding COVID-19–related myocardial injury and cardiogenic shock,^7,8^ the role of angiotensin-converting enzyme inhibitors in viral pathogenesis and cell entry,^9,10^ the concerning reduction in patients presenting with acute coronary syndromes,^11^ and the impact of the pandemic on cardiology training, academic productivity, and wellness across the field.^12,13,14,15^

The magnitude of the pandemic has simultaneously overshadowed and emphasized the importance of CVD prevention.^16,17^ As all providers were called to arms, cardiology’s pendulum swung even further toward reaction, intervention, and treatment and away from prevention, motivation, and counseling. But as mortality rates skyrocketed in the chronically ill, the U.S. was forced to reckon with its own health and ask difficult questions about why so many Americans have poor cardiovascular health. Obesity, diabetes mellitus, and CVD were no longer silent chronic diseases but risk factors for a deadly virus, thus elevating their prevalence in the national consciousness. Furthermore, the pandemic revealed not only the state of the nation’s health but also the state of its health care, with the burden of illness proportionally greatest among historically marginalized racial/ethnic minority groups^18^ who also experience poorer cardiovascular health, healthcare inequities, and structural racism.^19^ And while the pandemic exposed a healthcare system, it also exposed the field of cardiology, which too often prioritizes treating over preventing disease, reimburses for in-person procedures but not for telephone conversations about those procedures, and was largely unprepared for a pandemic of this scale and complexity.

With vaccines now widely available, we must mine the lessons of the pandemic to identify how healthcare delivery has changed and how it can improve to meet community demands during both pandemic and peacetime—with a special focus on the prevention of CVD and its risk factors. In this review, we discuss how the COVID-19 pandemic has disrupted cardiovascular care and how months of quarantine, isolation, and physical inactivity may now exacerbate the epidemic of CVD. We discuss what the pandemic has taught us about caring for patients who were most impacted—older adults, persons of color and other adults from historically marginalized communities, and those facing adverse socioeconomic circumstances—and who continue to be the most affected by CVD. We also identify opportunities for enhanced CVD prevention now boosted by the immediate adoption of telemedicine and other innovative cardiac care models. Lastly, we discuss how the COVID-19 pandemic motivated us to prioritize our health above all else and how we can leverage this increased health awareness and investment into long-term, meaningful disease prevention.

## Disruption of CVD Care During the Pandemic

At the advice of the Centers for Disease Control and Prevention and mandated by state and local governments, a large percentage of the population, particularly those with chronic illnesses, remained at home for much of 2020 and into 2021. This has led to significant, unintended reductions in inpatient and outpatient cardiac care. Across the country, admissions for acute coronary syndrome (as well as cath lab activations), heart failure, and stroke decreased dramatically.^20^ In the immediate period after the outbreak, in-person cardiology office visits dropped by over 60% in some areas of the country.^21^ Those patients who did make in-person visits presented significantly later in the course of a myocardial infarction, with studies showing more than a 3-fold increase in time from the onset of symptoms to revascularization.^22^ Primary care visits, which account for much of CVD prevention efforts, dropped more than 20% during the pandemic despite the broad adoption of telemedicine. Concerningly, studies showed a 50% reduction in blood pressure evaluations and 38% reduction in cholesterol level evaluations during the pandemic.^23,24^ Furthermore, cardiac rehabilitation programs were halted overnight, canceling millions of sessions, leading to staff reductions, and leaving patients without access to a resource that may reduce total and cardiovascular mortality by up to 30% compared with standard treatment.^25^

These concerning trends may continue throughout the long tail of the pandemic, depending largely on the success of vaccination efforts and effective patient education regarding the importance of seeking appropriate acute cardiovascular care when needed. It is critical that patients understand the need to seek care for chest pain or acute neurological symptoms, even in the context of lockdowns or social distancing measures, and cardiologists should optimize interactions with their most vulnerable patients by reinforcing this message. Additionally, cardiologists should discuss vaccine status (COVID-19 and influenza^26^) during each preventive cardiology visit, particularly for vulnerable populations who may hesitate to seek care.

For many of our most vulnerable patients, the COVID-19 vaccine could be one of the most important steps they take this year toward preventive cardiac care. Given the degree of misinformation surrounding vaccines, preventive cardiologists can play a pivotal role in helping to ground patients in the evidence regarding vaccine safety. Professional organizations promoting cardiovascular health, including the American Heart Association (AHA), can support preventive cardiologists by creating and routinely updating patient-centered educational materials regarding vaccine safety data and efficacy that can be shared during clinic visits. Aligning COVID-19 vaccination with patient goals for overall and cardiovascular health could help overcome vaccine hesitancy in some patients.

One of the numerous lessons from the disruption in cardiac care is the recognition of how rapidly the field can advance in response to such challenging circumstances. The overnight adoption of telemedicine for preventive cardiology is the most obvious example, but remarkable innovation took place across the field of cardiology. As elective coronary angiograms were stopped to maintain bed capacity and personal protective equipment, hospital systems reported a rapid adoption of coronary cardiac computed tomography as the primary means of evaluating chest pain in select patient populations.^27^ Although blood pressure evaluations dropped, studies showed that patients who enrolled in home blood pressure monitoring programs had no change in their frequency of measurements, highlighting the potential for such innovative programs.^28^ Furthermore, the Centers for Medicare and Medicaid Services (CMS) added virtual cardiac rehabilitation to the list of approved telehealth services, unlocking home-based programs for patients around the country.^29^

### Impact on Lifestyle and Implications for Cardiovascular Health

**Figure 1:**
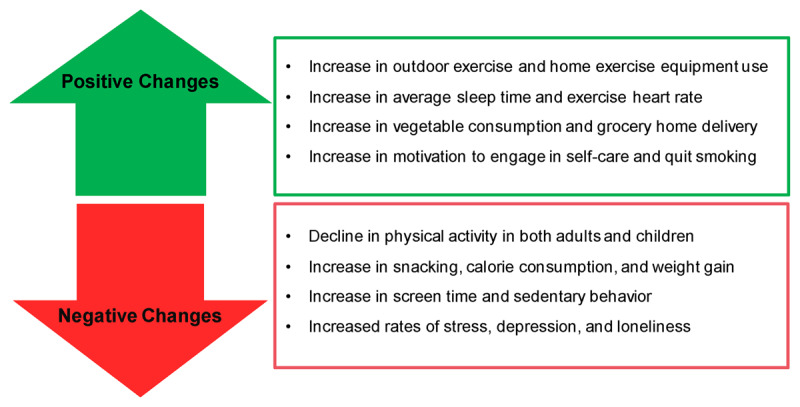
Summary of the positive and negative impact of COVID-19 on lifestyle and opportunities for healthier living in the post–COVID-19 world.

The aim of preventive cardiology is to align a patient’s cardiovascular health with their entire lifestyle, including their diet, exercise, sleep, family support, substance use, stress, screen time, and preventive medication. But when our lives were upended by the pandemic, so too were our preventive efforts (***Figure 1***). During most of 2020 and the first half of 2021, widespread quarantining and lockdowns led to a significant decline in physical activity both globally and nationally, with some regions seeing declines over 50%.^30^ Studies showed a significant decline in children as young as 5 years old, a pattern that could become entrenched and have negative long-term health consequences if not aggressively addressed.^31^ Diets changed even more dramatically, with 85% of people reporting changes to their eating habits or food preparation. With entire families at home, there was the potential for individuals to snack more, think about food more, and consume more calories than usual.^32,33^ A study of lifestyle activities in 7,753 participants found weight gain in 27.5% of total participants and in 33.4% of obese participants.^34^ With the dramatic increase in time spent at home, it is no surprise that screen time skyrocketed across televisions, tablets, and smartphones, further contributing to the rise in sedentary behavior.^35^

Increasing rates of stress, anxiety, depression, loneliness, and social isolation, all known risk factors for CVD, accompanied each of these disruptions.^36^ A study of patients presenting with acute coronary syndromes found 4.6 times the rates of stress-related cardiomyopathy during the early months of the pandemic compared with a recent pre-pandemic period.^37^ Each of these trends would be concerning on their own, but the pandemic evoked them all at once, challenging CVD prevention efforts during this period and potentially for years to come.

## The Pandemic and Reshaping Preventive Cardiology

**Figure 2:**
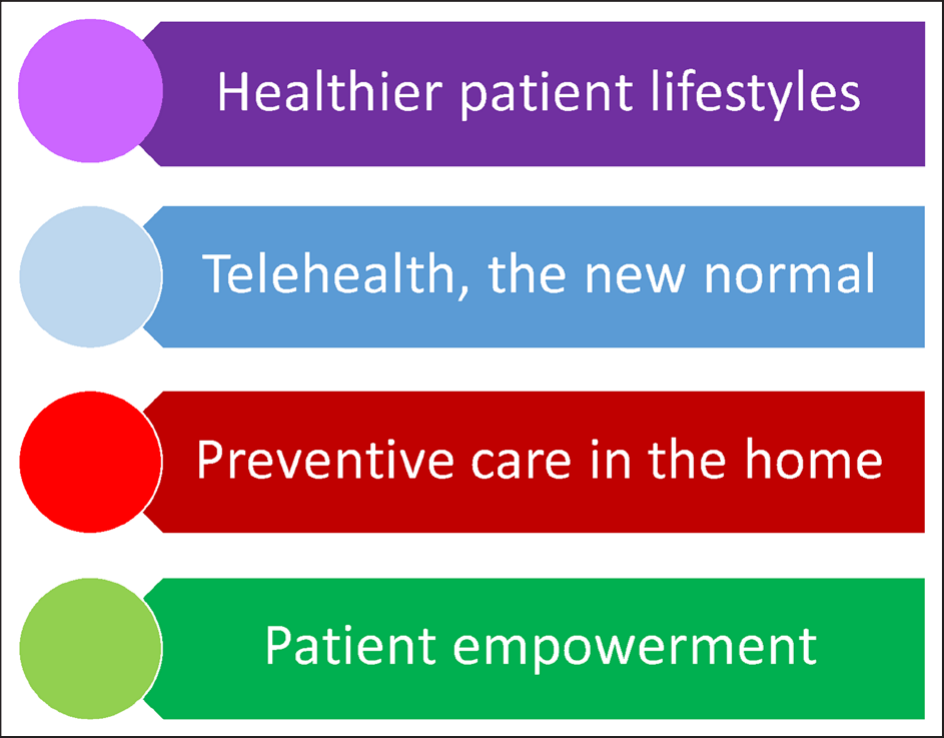
Four approaches that can reshape primordial cardiovascular prevention and preventive care, each one sharpened by the challenges of the pandemic.

As the healthcare system recovers in the wake of the pandemic, we now have a unique opportunity to shift away from inertia and determine what can be leveraged from this experience to improve preventive cardiology going forward. Leaders in the field have called for redefining all preventive care in this country in the aftermath of the pandemic.^38^ Within cardiology, as mortality rates have plateaued in recent years after decades of decline,^39^ we as healthcare providers should take this opportunity to do the same. Here we discuss four approaches that can reshape primordial cardiovascular prevention and preventive care, each one sharpened by the challenges of the pandemic (***Figure 2***).

### 1) Opportunities for Healthier Lifestyles

The lifestyle changes required by the pandemic created important opportunities for primordial and primary CVD prevention that can and should be continued going forward (***Figure 1***). For instance, despite the decline in step counts and the closure of gyms across the country, the pandemic also inspired a “fitness revolution” of new runners and home exercisers.^40^ The rise of fitness technology companies like Peloton revealed that not all screen time is sedentary.^41^ The data released by Fitbit showing a drop in step count also showed a significant drop in the average heart rate of users, which could be attributed to additional data the company released showing increased high-intensity exercise and increased sleep time throughout the pandemic.^42^

The same studies that showed increased weight gain and stress also showed increased rates of cooking at home,^34^ and the studies showing increased snack consumption also showed increased vegetable consumption.^33^ Furthermore, a respiratory virus pandemic represented the perfect time to motivate smoking cessation; studies showed that almost 20% of smokers smoked fewer cigarettes and one-third were more motivated to quit.^43^ The societal disruption caused by the pandemic could have led to the failure of multiple metrics—diet, exercise, stress, or substance use—that combine to drive CVD prevention. Fortunately, the pandemic revealed new avenues for CVD prevention within its constraints. An analysis of Google search patterns reflecting online shopping behavior early in the pandemic revealed that there was an interest in overall healthier CVD lifestyle changes, although this was not sustained.^44^ It is now the responsibility of societies, public health leaders, and healthcare providers to recognize those new opportunities and carry them forward into post-pandemic care.

### 2) Expanding Preventative Cardiology Telehealth Beyond the Pandemic: The New Normal

Given their potential benefits, the adoption of telehealth platforms for preventive cardiology visits could continue to evolve into the standard of care beyond the pandemic. Although total blood pressure and cholesterol evaluations declined, the field of cardiology experienced the greatest migration to telehealth platforms by volume of any field in medicine except for primary care.^45^ Hypertension, hyperlipidemia, and diabetes ranked first, second, and fourth, respectively, in the top indications for telehealth appointments in the early months of the pandemic.^45^ Preventive cardiology visits, which focus heavily on lifestyle improvements, medication adherence, and patient motivation, lend themselves uniquely well to a virtual visit. While a physical exam can be useful during the initial visit, similar information can be obtained by eliciting the weight, height, and abdominal circumference of the patient. Furthermore, telehealth platforms are well suited to leverage the multidisciplinary nature of preventive cardiology care: a single video visit can include sequential or simultaneous meetings with a physician to discuss starting a protein convertase subtilisin/kexin type-9 inhibitor, a nurse to provide instruction on self-administering the injection, and a social worker to evaluate insurance coverage.

For this to become a reality, the reimbursement landscape must support this approach. As the pandemic intensified, CMS added 85 telehealth services to the list covered by Medicare, with reimbursements at the same rate as in-person visits. This is progress, but likely only temporary, and when the pandemic subsides only 22% of states will have telehealth parity laws in place.^46^ Furthermore, telehealth platforms will need to be designed specifically for vulnerable patient populations to ensure they bridge rather than expand the digital divide. Families with income < $50,000 are significantly less likely to use video-based platforms for telehealth visits, the platforms of choice for most insurance companies.^47^ The staying power of telehealth will be determined by its ability to reach all families, connect them reliably with providers, and improve health outcomes.

### 3) CVD Preventative Care at Home

The COVID-19 pandemic confined patients to their homes for months on end and often required providers to manage sick patients remotely. Patients with COVID-19 were discharged from the emergency department with instructions to buy a pulse oximeter and follow up with their primary care physician. Cardiac patients were instructed to buy a home blood pressure cuff and a scale. This was a glimpse into the future of what chronic preventive cardiology care could look like—patients monitoring their own risk factors and established CVD from home, with providers interjecting when necessary. There are now remote algorithm-based cardiovascular risk management programs that adjust cholesterol, blood pressure, and even heart failure therapies, and attain significant improvement in outcomes without the direct involvement of a physician.^48,49^ However, equal energy focused on integrating these mobile health applications and remote monitoring systems into electronic medical records would help transform these new waves of patient data into actionable insight. More studies, as well as guidance from professional societies, are needed to help clinicians identify the best evidence-based mobile health technology (mHealth) approaches.

As discussed, the pandemic expedited the adoption of home-based cardiac rehabilitation. Additionally, it led to a spike in mail-order drug delivery, which jumped 21% during the first month of the pandemic.^50^ There are certainly many benefits of mail-order drug delivery, including a lower copay, 100-day medication supply with free shipping, and convenience online or by phone, with no need to drive to a pharmacy and wait in line every month. Preventive cardiologists should take note, as studies show that patients who receive their statin by mail may be more likely to remain adherent and see greater low-density lipoprotein (LDL)-cholesterol reduction than those who pick up that same statin from a pharmacy.^51^ In another study of stroke patients, users of mail-order pharmacy services were more likely to be adherent to preventive medications and had reduced hospital readmissions compared to those who made in-person pharmacy visits.^52^

Across the care continuum, from medications to remote monitoring and rehabilitation, the pandemic forced providers to meet patients in their homes. Within the comfort and safety of their own homes, patients may feel more relaxed to engage in preventive discussions outside of the typical office setting. A home environment also can provide additional opportunities, such as engaging partners or other social supporters about prevention, “touring” a patient’s pantry to provide nutrition counseling, reviewing medication labels to confirm a medication list and dosing, watching a patient take their own blood pressure with their home device to confirm adequacy of technique, and so forth. This paradigm shift, empowered by telehealth platforms, represents an exciting growth opportunity for the field if developed wisely in the post–COVID-19 world.

### 4) Patient Empowerment

**Figure 3:**
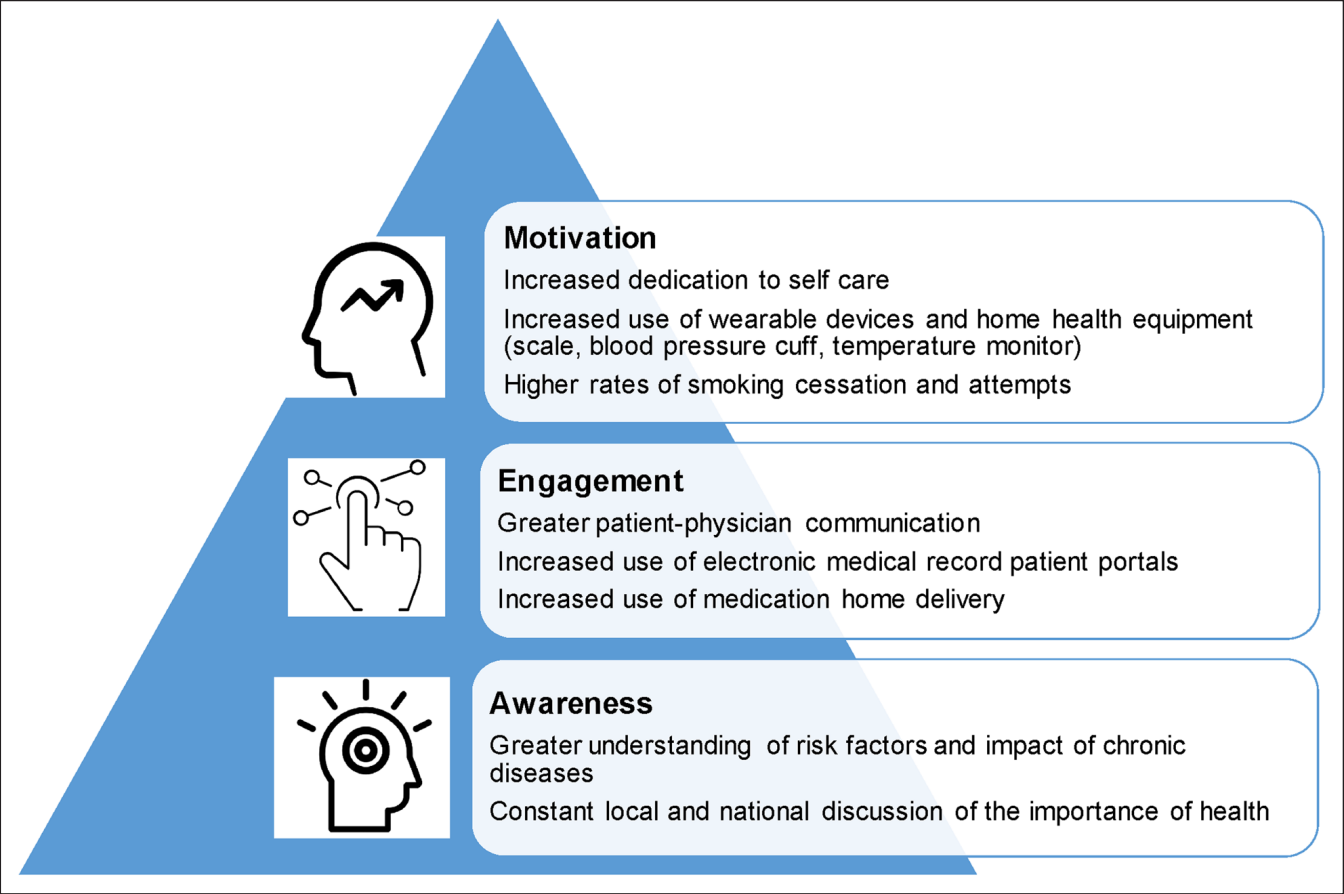
As demonstrated throughout the COVID-19 pandemic, the three ingredients for patient empowerment include awareness, engagement, and motivation.

The pandemic’s most important contribution to CVD care and prevention is not the expansion of telemedicine, new devices, or home fitness routines. While CVD providers must leverage all of these opportunities for enhanced disease prevention and care, the most important resource to leverage is the awareness, motivation, and empowerment of each individual patient to be healthy (***Figure 3***). This is the paramount lesson of the COVID-19 pandemic and one that aligns directly with the goals of CVD prevention providers. The investment that patients have made in their health, such as social distancing, mask-wearing, and getting vaccinated, can translate to diet, exercise, and medication adherence. Recent national surveys report that 80% of Americans intend to be more mindful about regular self-care practices after the pandemic, and over 40% of Americans desire more guidance and support for practicing self-care.^53^

In addition, patients’ increased engagement with personal health boosted their engagement with health systems; as questions about health multiplied, so too did activation of patient portal accounts and messages to providers. In California, the volume of patient-to-provider messages multiplied by over 28-fold over a 65-day period early in the pandemic.^54^ While physicians and public health officials recommended mask-wearing, social distancing, and vaccination, most patients heeded this advice on their own, without the need for monitoring or prescribing. This dynamic may be possible with CVD prevention, only it plays out over decades.

## Considerations for Vulnerable Populations

“The brunt of COVID-19 will be borne by the poor, elderly, and sick, and it is up to us to ensure they are not left behind,” said former U.S. Surgeon General Vivek Murthy.^55^ Findings from the AHA’s COVID-19 CVD Registry soberly demonstrated the disproportionate morbidity and mortality experienced by vulnerable populations in the pandemic.^18^ Minority communities had the highest rates of cardiovascular comorbidities, including hypertension, diabetes, and obesity, all of which have been associated with severe COVID-19 courses. The disproportionate burden of cardiovascular morbidity and mortality among Black, Indigenous, and people of color had been well established prior to COVID-19, but the pandemic acted as a stress test that unearthed the great deal of work to be done in cardiovascular prevention for these vulnerable groups.^19^

Importantly, the pandemic highlighted the socioeconomic issues that vulnerable communities face when seeking health care, such as limited access and housing and food insecurity.^56^ For women in particular, who already bear a disproportionate caregiving burden, the pandemic increased exposure to sick loved ones and, with the closure of schools and daycares, imposed barriers to participating in routine health visits and investing in personal well-being.^57^ In a study by Al Rifai in 2020, the prevalence of internet use in patients with atherosclerotic CVD was lower among racial/ethnic minority groups.^58^ We must be mindful that increased utilization of telemedicine and mobile health technology will likely widen the digital divide and create even greater inequities in access to care.

Improving cardiovascular prevention for vulnerable groups will depend in part on the collective efforts of the cardiology community to dismantle systemic barriers. Advocacy from professional organizations, such as the Association of Black Cardiologists (ABC), should be supported and emulated. For example, the ABC is modeling community-based interventions, such as their Release the Pressure campaign, to address hypertension in Black women through collaboration with key organizations, including churches and community centers; this is modeled after similar successful interventions addressing hypertension in Black men by partnering with local barbershops.^59^ The AHA recently outlined its commitment to mitigating the effect of structural racism on cardiovascular health by investing in research and community programs^19^ and identified health equity as a guiding principle for its 2030 Impact Goal to increase longevity.^60^ The cardiology community must leverage the collective voice of these organizations to lobby for meaningful policy changes.

While organizations advocate for change in the public sphere, providers can seize opportunities to improve cardiovascular care for vulnerable groups in the clinic setting. Such opportunities include increasing use of mail-order pharmacies to improve access to preventive therapies, expanding use of telehealth for communities with limited transportation, offering flexible clinic schedules to accommodate essential workers and those with childbearing responsibilities, promoting access to technology such as home blood pressure cuffs to titrate therapy, and investing in social and community health workers within cardiology clinics to connect patients with resources that address housing and food insecurity. Cardiology clinics should consider investing in interpreter services that support the large expansion of telemedicine to ensure that non–English-speaking patients can effectively access care via these novel platforms rather than face additional barriers.

Although COVID-19 will pass, structural barriers mediating cardiovascular health will persist, and the cardiology community must recognize its role in closing the CVD morbidity and mortality gap.

### Older Adults

Social distancing emerged as a core public health strategy and is expected to remain a mainstay of future pandemic responses. However, COVID-19 illustrated the disproportionate toll of social isolation on older adult populations. Long before this pandemic, social isolation in older adults has been linked to CVD, increasing the risk of hypertension, coronary artery disease, stroke, heart failure, and all-cause mortality.^61^ While the pandemic’s effect on adults of advanced age are yet to be fully characterized, already higher rates of CVD are being observed among isolated populations.^62^

The AHA recently published a scientific statement emphasizing the interconnectivity of the mind, heart, and body and focusing on the “whole person” to improve cardiovascular health.^63^ Indeed, solutions to mitigate the negative impact of social isolation on older adults have emerged from different sectors. From the technology sector, digital solutions such as teleconference platforms connected older adult patients with loved ones.^64^ Community organizations bridged the void where digital solutions fell short, enlisting volunteers to call elderly patients and organizing socially distant outdoor events.^65^ Healthcare models protocolized screening to link at-risk older patients with community resources and reimbursed services connecting seniors with companions who monitored medication adherence.^66^ While COVID-19 magnified the pre-existing association between loneliness and CVD among geriatric patients, it highlighted the need for cardiologists to become familiar with emerging services to support seniors.

Additionally, social isolation erected significant barriers to exercise, with older adults at highest risk of deconditioning and falls. Adaptation of exercise regimens, validated for hospitalized seniors to home settings, can empower older patients to maintain physical well-being.^67^ The momentum of home and virtual fitness among older adults should be seized by cardiologists to improve prevention and cardiopulmonary fitness in the years to come.

The care delivery landscape changed dramatically with use of telehealth among US seniors, which increased 10-fold in 2020.^68^ The pandemic proved that telehealth can be a viable strategy to improve care access for seniors. Nonetheless, more must be done to align technology with geriatric needs. With adults over 65 projected to represent 20% of the US population by 2030 and outnumber children for the first time in history,^69^ emerging technology must be informed by the unique audiovisual, physical, and cognitive needs of seniors. Cardiologists should seek opportunities to integrate evidence-based remote patient monitoring devices such as ambulatory blood pressure cuffs, smartphone-electrocardiograms, and wearable glucose monitors into the preventive care of geriatric patients.^64^ Although less than a third of seniors reported telehealth encounters with specialists, there is an opportunity for cardiologists to expand their digital footprint in a population that has proven itself more than willing and capable of engaging with telemedicine.^68^

## Conclusions

When reviewing CVD prevention throughout the COVID-19 pandemic, the first tendency may be to focus on the negative disruption: visits missed, care diverted, medications left unfilled. It is easy to imagine 2020, a year in quarantine before vaccinations, as a lost year or a step backward for many of our patients, a time when preventing immediate disease superseded preventing chronic disease. CVD prevention is an uphill battle against structural forces in our society and the unknowable complexities of our patients’ lives. Looking back, we should see not a lost year but a pandemic that better prepared us for future battles, with new platforms for interacting with patients who are more engaged than ever. We should see a unique opportunity to reset preventive care in this country. In doing so, we can adjust the course of CVD away from a pro-obesogenic society and toward a new normal that prioritizes the prevention of disease and empowers patients to live their healthiest lives.

## Key Points

- Although much attention and healthcare resources were appropriately diverted to managing the COVID-19 pandemic, cardiologists must refocus on efforts devoted to cardiovascular disease (CVD) prevention to battle the pandemic of cardiometabolic diseases.
- COVID-19 caused negative disruptions to CVD prevention, including (1) reduced physical activity; (2) increased snacking, calorie consumption, and weight gain; (3) increased screen time and sedentary behavior; (4) increased rates of social isolation, mental stress, depression, and loneliness; and (5) missed clinic visits, resulting in delayed acute and preventive care.
- The COVID-19 pandemic also spurred favorable changes in CVD prevention, including (1) increases in the use of home exercise equipment, sleep time, vegetable consumption, and home grocery delivery; (2) rapid adoption of telehealth; (3) greater patient-physician communication; (4) increased use of medication home delivery; (5) greater understanding of risk factors and impact of chronic diseases; and (6) motivation to engage in self-care.
